# On the holobiont ‘predictome’ of immunocompetence in pigs

**DOI:** 10.1186/s12711-023-00803-4

**Published:** 2023-05-01

**Authors:** Joan Calle-García, Yuliaxis Ramayo-Caldas, Laura M. Zingaretti, Raquel Quintanilla, María Ballester, Miguel Pérez-Enciso

**Affiliations:** 1 Centre for Research in Agricultural Genomics CSIC-IRTA-UAB-UB, Campus UAB, Edifici CRAG, 08193 Bellaterra, Spain; 2 Animal Breeding and Genetics Program, Institut de Recerca i Tecnologia Agroalimentàries (IRTA), Caldes de Montbui, 08140 Barcelona, Spain; 3 ICREA, Passeig Lluis Companys 23, 08010 Barcelona, Spain; 4 Present Address: Corteva Agriscience, Virtual Location, Bergen op Zoom, Indianapolis, 4611 BB Netherlands

## Abstract

**Background:**

Gut microbial composition plays an important role in numerous traits, including immune response. Integration of host genomic information with microbiome data is a natural step in the prediction of complex traits, although methods to optimize this are still largely unexplored. In this paper, we assess the impact of different modelling strategies on the predictive capacity for six porcine immunocompetence traits when both genotype and microbiota data are available.

**Methods:**

We used phenotypic data on six immunity traits and the relative abundance of gut bacterial communities on 400 Duroc pigs that were genotyped for 70 k SNPs. We compared the predictive accuracy, defined as the correlation between predicted and observed phenotypes, of a wide catalogue of models: reproducing kernel Hilbert space (RKHS), Bayes C, and an ensemble method, using a range of priors and microbial clustering strategies. Combined (holobiont) models that include both genotype and microbiome data were compared with partial models that use one source of variation only.

**Results:**

Overall, holobiont models performed better than partial models. Host genotype was especially relevant for predicting adaptive immunity traits (i.e., concentration of immunoglobulins M and G), whereas microbial composition was important for predicting innate immunity traits (i.e., concentration of haptoglobin and C-reactive protein and lymphocyte phagocytic capacity). None of the models was uniformly best across all traits. We observed a greater variability in predictive accuracies across models when microbiability (the variance explained by the microbiome) was high. Clustering microbial abundances did not necessarily increase predictive accuracy.

**Conclusions:**

Gut microbiota information is useful for predicting immunocompetence traits, especially those related to innate immunity. Modelling microbiome abundances deserves special attention when microbiability is high. Clustering microbial data for prediction is not recommended by default.

**Supplementary Information:**

The online version contains supplementary material available at 10.1186/s12711-023-00803-4.

## Background

Immunocompetence is an important contributor to productivity, profitability, and welfare in pigs as in other species [1, 2]. The current emergence of antibiotic resistance [3], coupled with increasing social demands for healthier products and environmentally responsible livestock systems, favour the incorporation of health-related traits in pig breeding programs [4]. Importantly, the relevance of the pig as a biomedical model is rapidly increasing, given its similarities with humans in terms of anatomy and physiology [5, 6].

The importance of the composition of human and animal microbial symbionts for health and production is now widely recognized [7–11]. In this context, the ‘hologenome’ concept [12], which describes the joint action of host genome and microbiome on a phenotype, is becoming increasingly popular in the literature. Since the pioneering work of Ross et al. [13], in which human health-related traits and methane production of a cow were predicted using microbiota information, numerous studies have used the composition of gut microbial ecosystems to elucidate its contribution to different complex animal traits, such as methane emission in cattle [13–15], carcass traits in pigs [16, 17], and feed efficiency in several farm animals [18–22], among others. The relevance of gut microbiota composition in the mammalian immune system has received special attention in the past years [10, 23]. Several studies have reported associations between microbial abundances and genomic polymorphisms in immunity-related genes [24–27], which is in line with previous reports stating that the host genome can influence the composition of the gut microbiota [26, 28–30]. In a recent study [9], we explored the contribution of the host’s genotype and its gut microbiota to several immunocompetence traits in a Duroc pig population, and we found an important connection between gut microbiota composition and pig immunity.

Considering the contribution of microbial composition to animal physiology and immunity, the next natural step is to incorporate this information into a prediction framework. However, guidelines for the integration of microbial abundances with the currently ubiquitous genotype marker data are lacking. The analogy between microbiome and genotype marker data, i.e., that the number of features typically exceeds the number of samples, should not hide some important differences between both types of data: (i) microbiota composition may change throughout the life of an organism [31], (ii) there are many more microbial genes than genes in the host genome [32], (iii) the microbiome has a hierarchical structure given by its phylogenetic relationships, (iv) microbial abundance distributions are zero-inflated and highly leptokurtic, and (v) microbial abundances are compositional, which induces relationships between abundances [33].

Using the holobiont, i.e., genotype and microbiome data, to better understand and predict complex traits is still largely unexplored. Some studies have integrated genotype and microbiota composition data in the same model to explore their contributions to complex traits [18, 34–36], while genetic and microbial parameters were independently analysed in other studies [15, 21, 22, 37].

In this study, and complementing our previous simulation work [38], we evaluated the performance of alternative modelling strategies for the prediction of immunocompetence in pigs. The diversity of the genetic and microbial influences on immunocompetence in pigs that has been reported in our previous work [9] makes immunocompetence traits a good case study for exploring the impact of different holobiont modelling strategies. Here, we focus on prediction and explore a wide range of choices in terms of statistical models, priors, and abundance clustering. We named this large catalogue of model choices for prediction the ‘predictome’.

## Methods

### Animal samples

The animal material used in this study belonged to a commercial Duroc line and is fully described in Ramayo-Caldas et al. [9]. The animals used here were a subset of 400 Duroc piglets (199 females and 201 males) out of the 432 in [9]. They were the offspring of 22 boars and 132 sows. Animals were raised on the same farm, fed ad libitum with a commercial cereal-based diet. They were apparently healthy, without any sign of infection. Blood and faecal samples were collected at 60 ± 8 days of age in six different batches (dates). Fecal samples were transferred to cryotubes, conserved in ice, and later stored at − 80 °C until DNA extraction.

### Immunocompetence traits

For this study, we selected six phenotypes that covered a range of genetic and microbial parameters based on previous evidence [9]. These phenotypes were classified according to the two major components of the immune system: plasma concentrations of immunoglobulins M (IgM) and G (IgG), which are associated with adaptive immunity, and serum concentrations of the acute-phase proteins haptoglobin (HP) and C-reactive protein (CRP) plus the phagocytic capacity of lymphocytes (LYM_PHAGO_FITC), which are components of innate immunity. Finally, the percentage of gamma-delta T cells (γδ T cell), which are a bridge between innate and adaptive immunity, was also considered. Details of the sampling and laboratory processing are in [9]. All traits, except immunoglobulins (IgM and IgG) had a highly leptokurtic distribution, which was addressed by log-transformation. Data were preadjusted prior to cross-validation to focus on genetic and microbial effects rather than on environmental factors. Following previous analyses [39], IgM, IgG, CRP, and γδ T cell phenotypes were corrected for the effect of batch (sampling date), HP was corrected for the effects of batch and sex, and LYM_PHAGO_FITC was corrected for the effects of batch and sex.

### Genotype data

Animals were genotyped for 68,516 single nucleotide polymorphisms (SNPs) with the Porcine 70 k GGP Porcine HD Array (Illumina, San Diego, CA, USA), using the Infinium HD Assay Ultra protocol (Illumina). We used the Plink v1.9 software [40, 41] to remove SNPs that had a minor allele frequency less than 5%, that had more than 10% missing genotype data, that mapped to the sex chromosome, or that did not map to the porcine reference genome (Sscrofa11.1 assembly). After quality control, 41,131 SNPs were retained for subsequent analysis. Missing genotypes were rare (0.19%) and were simply imputed with the average allele frequency of each SNP.

### Microbiota abundance data

To increase the read depth of microbial abundances available in Ramayo-Caldas et al. [9] and increase the reliability of the experiment reported here, we combined 16S metagenome data from [9] with new sequence data obtained from the same DNA samples. The bioinformatic procedure was the same and a strict quality control was applied to ensure the data were comparable, as described below.

DNA extraction was carried out with the DNeasy PowerSoil Kit (QIAGEN, Hilden, Germany), following manufacturer’s instructions. The first sequence dataset was obtained with paired end NovaSeq (2 × 250 nucleotides), while the second sequence batch was obtained with paired end Illumina MiSeq (2 × 300). The same primers were used for both batches: (V3_F357_N) 5′-CCTACGGGNGGCWGCAG-3′ and (V4_R805) 5′-GACTACHVGGGTATCTAATCC-3′.

Each 16S sequence dataset was processed independently with the QIIME2 v 2021.8 software [42], using the same bioinformatic pipelines. Denoising [43–46] was performed to extract each amplicon sequence variant (ASV) from the raw sequencing data using the R package dada2 [44], as implemented in the denoise-paired QIIME2 plugin. Primers were manually removed in both batches by trimming out the first 17 and 21 nucleotides from the forward and reverse strands, respectively. No truncation was performed on the first sequencing batch, but both forward and reverse sequences were truncated to 250 nucleotides in the second batch due to low quality. The two batches were merged after denoising with the feature-table merge plugin and the overlap method ‘sum’. The raw number of ASV was 57,195, but ASV that were present in less than three samples and that represented less than 0.001% of the total counts were discarded. These cut-offs are within the optimal range suggested by [47]. Centered log-ratio (CLR) transformation was applied to raw ASV abundances for further analyses. The pipeline used to process and combine these datasets is in Additional file 1.

Abundances were considered either by ASV or clustered, the latter in order to evaluate the effect of reducing the number of variables in the model. Two clustering options were tested: by phylogeny and by abundance. For taxonomic assignment, a classifier was created with QIIME2 v 2021.8 using the GreenGenes version 13.8 database [48]. For phylogeny clustering, the ASV sequences that passed quality control were aligned using the MUSCLE algorithm [49] and the output was used to perform the phylogeny analysis with the UPGMA algorithm [50]. Alignment and phylogeny analyses were conducted using MEGA version 11 [51]. The resulting rooted phylogeny tree was processed with the cutree function of the stats R package [52]. Since not all ASV could be assigned to the genus level and not all genuses collapsed at the same height of the phylogeny tree, the tree was sliced at different heights (h), and the consistency in the taxonomic composition at the genus level of the ASV in each subtree was evaluated. We found height h = 0.6 to be the optimum value. The leaves, i.e., the ASV, of each subtree were clustered, yielding k = 232 phylogeny-based clusters that corresponded approximately to the genus levels. The hclust package with Ward’s [53] method was used for abundance clustering. The cutree function was used again to generate the number of desired clusters. We used k = 232 to obtain the same number of clusters as in the phylogeny approach and facilitate comparison between clustering methods.

### Prediction analysis

We compared the predictive accuracy of Bayesian reproducing kernel Hilbert space (RKHS) regression and Bayes C [54] models implemented with the BGLR R package [55]. In Bayes C, the combined, holobiont model (referred to as model XB) was:

1a $$\mathbf{y}=\mathbf{m}+\mathbf{X}{{\varvec{\upbeta}}}_{\mathbf{G}}+\mathbf{B}{{\varvec{\upbeta}}}_{\mathbf{B}}+\mathbf{e},$$

where $\mathbf{y}$ is the corrected phenotype vector of size n, the number of samples (400); $\mathbf{m}$ is the general mean; $\mathbf{X}$ is the matrix of standardized SNP genotype value; $\mathbf{B}$ is the standardized CLR-transformed matrix of microbial abundances, either individual ASV or clusters; ${{\varvec{\upbeta}}}_{\mathbf{G}}$ and ${{\varvec{\upbeta}}}_{\mathbf{B}}$ are the corresponding vectors of SNP and ASV effects, respectively; and $\mathbf{e}$ is the vector of residuals. Prior to standardization, SNPs were originally coded as 0, 1, 2 for ‘*AA*’, ‘*AB*’ and ‘*BB*’ genotypes, respectively. Partial models that considered only genotypes (model X) or ASV abundances (model B) were also evaluated:

1b $$\mathbf{y}=\mathbf{m}+\mathbf{X}{{\varvec{\upbeta}}}_{\mathbf{G}}+{\mathbf{e}},$$

1c $$\mathbf{y}=\mathbf{m}+\mathbf{B}{{\varvec{\upbeta}}}_{\mathbf{B}}+{\mathbf{e}}.$$

In the case of the kernel-based method RKHS, the holobiont and partial models were:

2a $$\mathbf{y}=\mathbf{m}+\mathbf{Z}\mathbf{u}+\mathbf{W}\mathbf{b}+{\mathbf{e}},$$

2b $$\mathbf{y}=\mathbf{m}+\mathbf{Z}\mathbf{u}+{\mathbf{e}},$$

2c $$\mathbf{y}=\mathbf{m}+\mathbf{W}\mathbf{b}+{\mathbf{e}},$$

respectively, where $\mathbf{Z}$ and $\mathbf{W}$ are incidence (here identity) matrices for genotype and microbial abundances, respectively; $\mathbf{u}$ and $\mathbf{b}$ are vectors with genotypic and microbial random effects; and $\mathbf{e}$ is the vector of residuals. We assumed $\mathbf{u}\sim N(0,\mathbf{G}{\sigma }_{g}^{2})$ and $\mathbf{b}\sim N(0,\mathbf{M}{\sigma }_{b}^{2})$, where $\mathbf{G}=\frac{{\varvec{XX}}^{\prime}}{{\varvec{n}}_{\varvec{G}}}$ and $\mathbf{M}=\frac{{\varvec {BB}}{^\prime}}{{\varvec{n}}_{\varvec{B}}}$ are the genomic and microbial relationship matrices, respectively, ${n}_{G}$ being the number of SNPs (41,131) and ${n}_{B}$, the number of ASV (2945).

We compared a range of priors for both abundances and genotypes. In Bayes C, we assessed varying prior probabilities π0 of a feature (SNP, ASV or cluster) to enter the model. For SNPs, the values used were π0 = 0.5, 0,01, 0.001, and 0.0001; for individual ASV, π0 = 0.5, 0.1, 0.01, and 0.001; and for ASV clusters, π0 = 0.5, 0.1 and 0.01. In the case of RKHS, default prior parameters for variance components were taken as informative (referred to as FALSE), whereas an uninformative REML-like prior (referred to as TRUE) was achieved with parameters df0 = 0.0001 and S0 = 0.0001, where df0 is the degrees of freedom and S0 is the scale parameter of the variance component.

BGLR allows each feature, genotypes or abundances, to be modelled independently; thus we considered several modelling combinations: the mentioned methods and priors under both partial and combined models. Abundances were considered either at the individual ASV level or clustered. In total, 133 different models were evaluated for each trait. In addition, we also compared an ensemble method, computed as the average predicted values from all analyses for that trait [56].

To evaluate the accuracy of predictions, three partitions of 80 (20%) samples were removed and predicted using the model that was trained with the remaining data. Partitions were randomly chosen but keeping a fixed proportion of samples within each of the sampling batches. The correlation between observed and predicted phenotypes, averaged over the three partitions, was used as a measure of predictive accuracy. In each analysis, BGLR was run for 100k iterations, including 500 burn-in iterations, and thinning every five iterations. This number of iterations seemed sufficient for convergence (see Additional file 2: Fig. S1).

### Estimation of heritability and microbiability

We estimated heritability (h^2^) and microbiability (b^2^) using all combinations of priors and models described, using now the complete dataset. For RKHS, variance components are explicitly defined. For the Bayes C models, we used the approach suggested in [55] (https://github.com/gdlc/BGLR-R/blob/master/inst/md/heritability.md) to estimate heritability and microbiability. In short, at each iteration i, the method samples the effects of SNPs and ASV:

3 $${\mathbf{u}}_{\left(\mathbf{i}\right)}={\mathbf{X}{{\varvec{\upbeta}}}_{\mathbf{G}}}_{\left(\mathbf{i}\right)},$$

4 $${\mathbf{b}}_{\left(\mathbf{i}\right)}=\mathbf{B}{{\varvec{\upbeta}}}_{\mathbf{B}\left(\mathbf{i}\right)},$$

where ${\mathbf{u}}_{\left(\mathbf{i}\right)}$ and ${\mathbf{b}}_{\left(\mathbf{i}\right)}$ are sampled genome and microbiota effects at the $\mathrm{i}$-th iteration for the set of individuals, respectively. Therefore, ${\mathrm{h}}_{(\mathrm{i})}^{2}=\mathrm{Var}({\mathbf{u}}_{\left(\mathbf{i}\right)})/\mathrm{Var}(\mathbf{y})$ and ${\mathrm{b}}_{(\mathrm{i})}^{2}=\mathrm{Var}({\mathbf{b}}_{\left(\mathbf{i}\right)})/\mathrm{Var}(\mathbf{y})$ are the sampled h^2^ and b^2^ in the $\mathrm{i}$-th iterate, from which posterior means were estimated by averaging over iterations.

In addition, we computed the contribution of each ASV to total microbiability by setting the estimated effects for all ASV to zero except the $\mathrm{ASV}$ of interest. This was done using the modelling combination that yielded the highest predictive accuracy and that used Bayes C to model microbial abundances. We also estimated the heritability of the abundance of each ASV using RKHS.

## Results

### Quality control

Several quality control measures were applied to ensure that 16S reads from the two sequencing batches could be merged: (i) Euclidean distances between samples for each pair of datasets were highly correlated (Additional file 3: Table S1), (ii) samples showed no structure in a principal component analysis (PCA), neither for individual datasets nor for the merged dataset (see Additional file 2: Fig. S2) and (iii) at least 76% of ASV detected in the post-denoising combined dataset were also detected in each individual batch, and the number of detected ASV was similar for the two batches: 2971 vs. 2566 for batches 1 and 2, respectively. In the merged dataset, 2945 ASV pertaining to 53 genera were detected, of which 54% are classified at the genus level. In this dataset, the average number of 16S reads per sample was 136,616 (Additional file 2: Fig. S3). As expected, the distribution of ASV frequencies was highly leptokurtic-over 75% of the microbial ASV were present in only 124 samples or less (Additional file 2: Fig. S4).

### Predictive accuracies

**Fig. 1:**
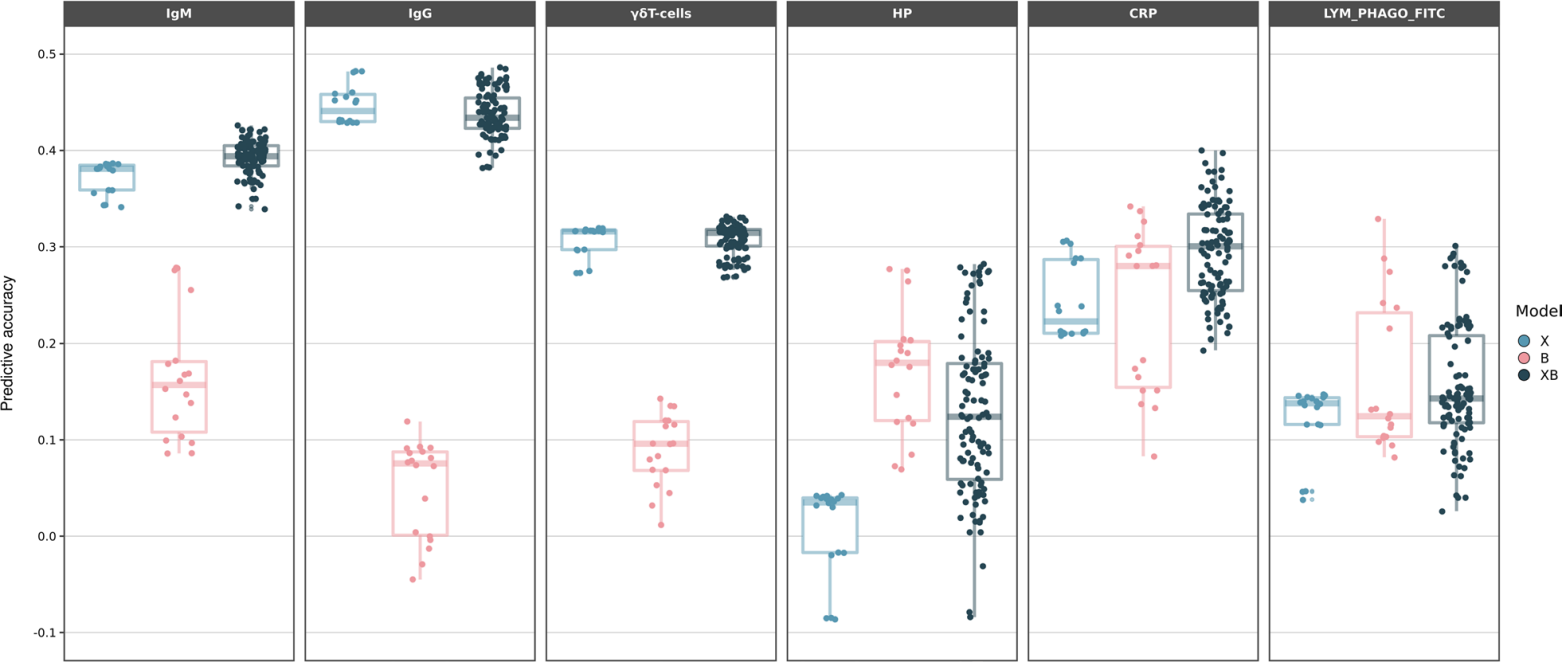
Predictive accuracy for each model and prior combination considered. Predictive accuracy, defined as correlation between predicted and observed phenotypes for each model, averaged over the three partitions. Models are classified by data used: SNPs only (Model X, Eqs. (1b) and (2b)), ASV only (Model B, Eqs. (1c) and (2c)), both SNPs and ASV (Model XB, Eqs. (1a) and (2a)). Each dot corresponds to the prediction accuracy obtained with a different combination of statistical method and prior, averaged over the three partitions

Predictive accuracies across models are shown in Fig. 1. Each dot corresponds to a predictive accuracy obtained with a different combination of statistical method and prior, averaged over the three partitions (see “Methods”). The relevance of genotypes and microbiota in prediction varied. We found genotype information to be more relevant than microbial abundances for predicting IgM and IgG and for the proportion of γδ T cells. In contrast, genotype data was not relevant for predicting HP. For CRP, both sources of information improved prediction when considered jointly: the best partial models X (Eqs. (1b) and (2b)) and B (Eqs. (1c) and (2c)) yielded similar predictive accuracies, which notably increased with the combined XB models (Eqs. (1a) and (2a)). Nevertheless, the best combined model was the best strategy for all traits, except for a single analysis under a partial microbiota model (B) for LYM_PHAGO_FITC. However, far more interesting is the observation that variability in predictions, i.e., ‘sensitivity’ to modelling, of models that include microbiota information was larger for traits for which the microbiome was more relevant (HP, CRP and LYM_PHAGO_FITC) than for traits for which genotypes sufficed for prediction (immunoglobulins and γδ T cells).

**Fig. 2:**
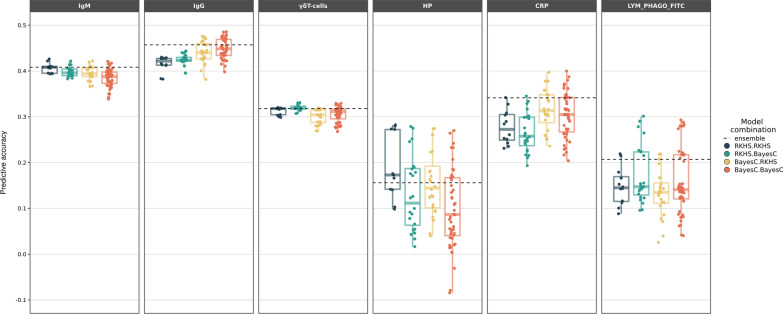
Predictive accuracy for each model in holobiont models. Predictive accuracy, defined as correlation between predicted and observed phenotypes for each model, averaged over the three partitions. The first element of each combination is the method (Bayes C or RKHS) used to model the genotype, while the element after the dot is the method used to model microbiota abundance. Each dot corresponds to the prediction accuracy obtained with a different combination of statistical method and prior, averaged over the three partitions. The dashed line is the predictive accuracy of the ensemble method

Next, we investigated the impact of RKHS and Bayes C on prediction using different combinations of holobiont models (Fig. 2). No method was uniformly best across all traits. However, it is relevant to note that the ensemble method was consistently better, or very similar in the worst case, than the average performance of the two individual methods. Beyond that, no clear pattern emerged.

**Fig. 3:**
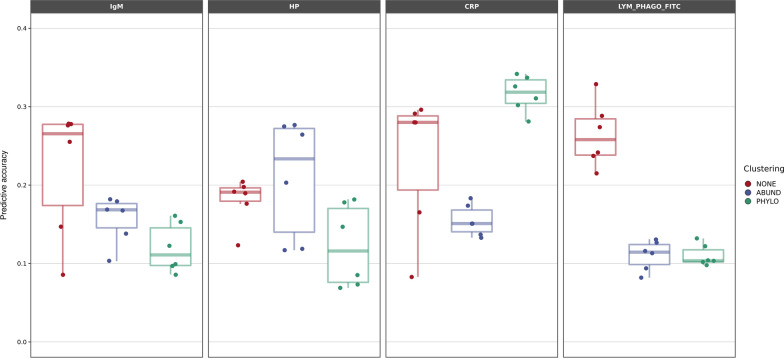
Predictive accuracy with microbiome clustering for the partial microbiota model. Predictive accuracy, defined as correlation between predicted and observed phenotypes for each analysis and trait, averaged over the three partitions, for each clustering approach: NONE, microbiota at the ASV level; ABUND, microbiota clustered by abundance; PHYLO, microbiota clustered by phylogeny. Results for IgG and γδ T cells are not shown since their prediction accuracy was not influenced by the microbiome (Fig. 1). Each dot corresponds to the prediction accuracy obtained with a different combination of statistical method and prior, averaged over the three partitions

Given that abundance distributions were highly leptokurtic (Additional file 2: Fig. S4), it is pertinent to ask whether clustering ASV in fewer groups could improve prediction. Figure 3 shows prediction accuracies when all 2945 ASV were included in the model individually, clustered at the genus level or by abundance. For the latter two cases, the number of clusters was k = 232. Only traits for which microbial abundances were relevant for prediction (Fig. 1) were considered in this comparison, i.e., IgM, HP, CRP, LYM_PHAGO_FITC. Clustering had an important effect on prediction accuracy for all traits, although not always in a positive direction. Clustering by abundance improved prediction for HP and by phylogeny for CRP concentration. Clustering worsened prediction for the remaining traits, especially for LYM_PHAGO_FITC.

### Impact of modelling on heritability and microbiability estimates

**Fig. 4:**
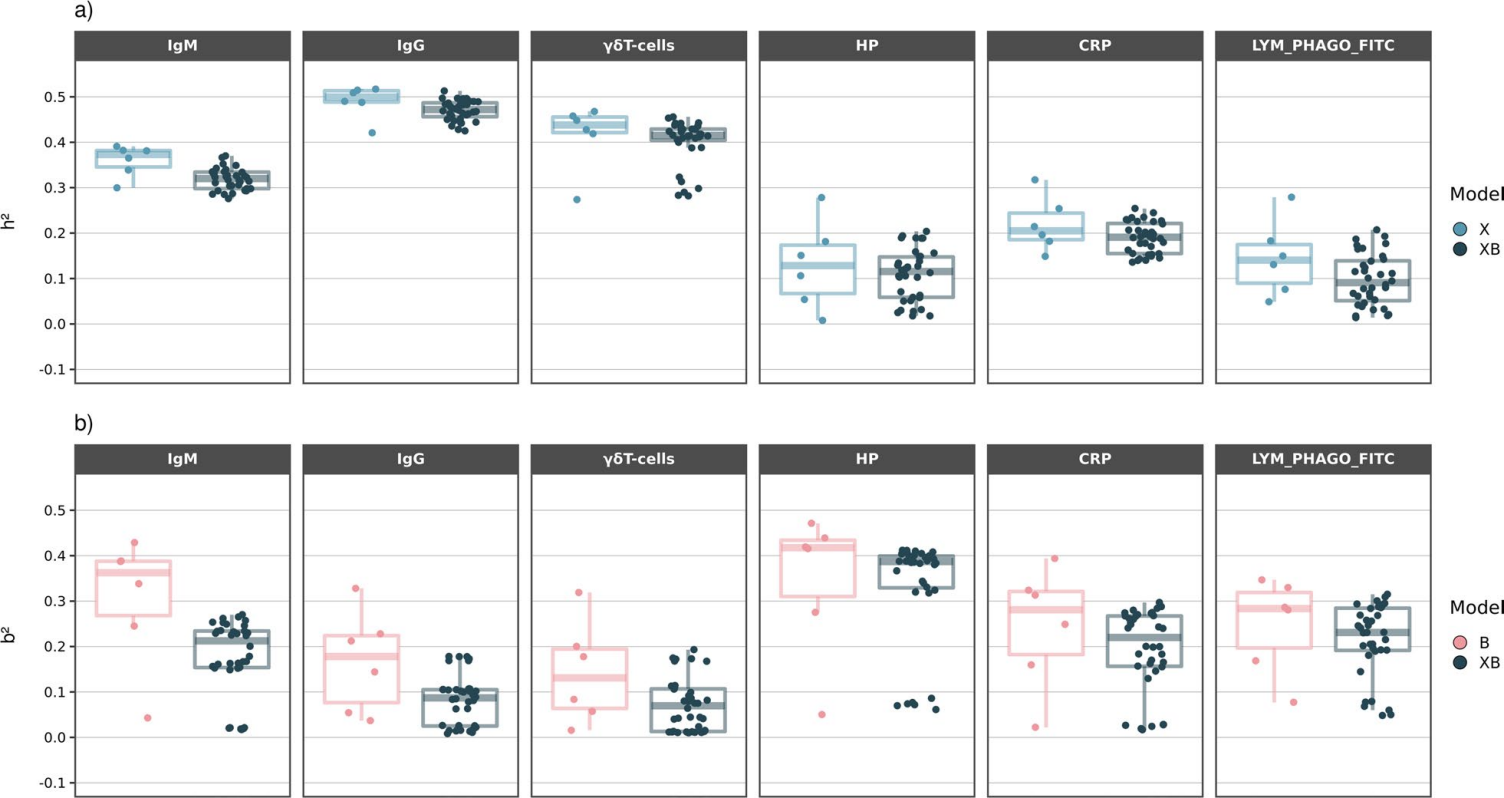
Distribution of estimates of heritability (**a**) and microbiability (**b**) obtained from partial and combined models. Estimates were obtained from the whole dataset. Model X: only genotypes are used (Eqs. (1b) and (2b)); model B: only abundances are used (Eqs. (1c) and (2c)); model XB: genotypes and abundances used (Eqs. (1a) and (2a)). Analyses with clustered microbial abundances were excluded. Each dot corresponds to an estimate obtained with a different combination of statistical method and prior in the whole dataset

Different modelling approaches are expected to be reflected in estimates of h^2^ and b^2^. Furthermore, although the ‘true’ model is never known, it is useful to know how sensitive estimates of h^2^ and b^2^ are to alternative models, and what are the estimates that correspond to the best models in terms of prediction. Unsurprisingly, given the prediction results (Fig. 1), estimates of h^2^ were much larger than estimates of b^2^ for IgG and γδ T cells, while the opposite was observed for HP (Fig. 4). Estimates of h^2^ and b^2^ obtained from the holobiont (XB) model were comparable in the case of CRP. We observed a large variability in estimates across modelling combinations, especially for b^2^. Overall, larger estimates of either h^2^ or b^2^ were obtained with the simple X or B models than with the complete holobiont models. This trend was far more marked for the estimates of b^2^ and suggests some confounding between genotype and microbiome effects.

### Effect of microbial abundances on immunocompetence

**Fig. 5:**
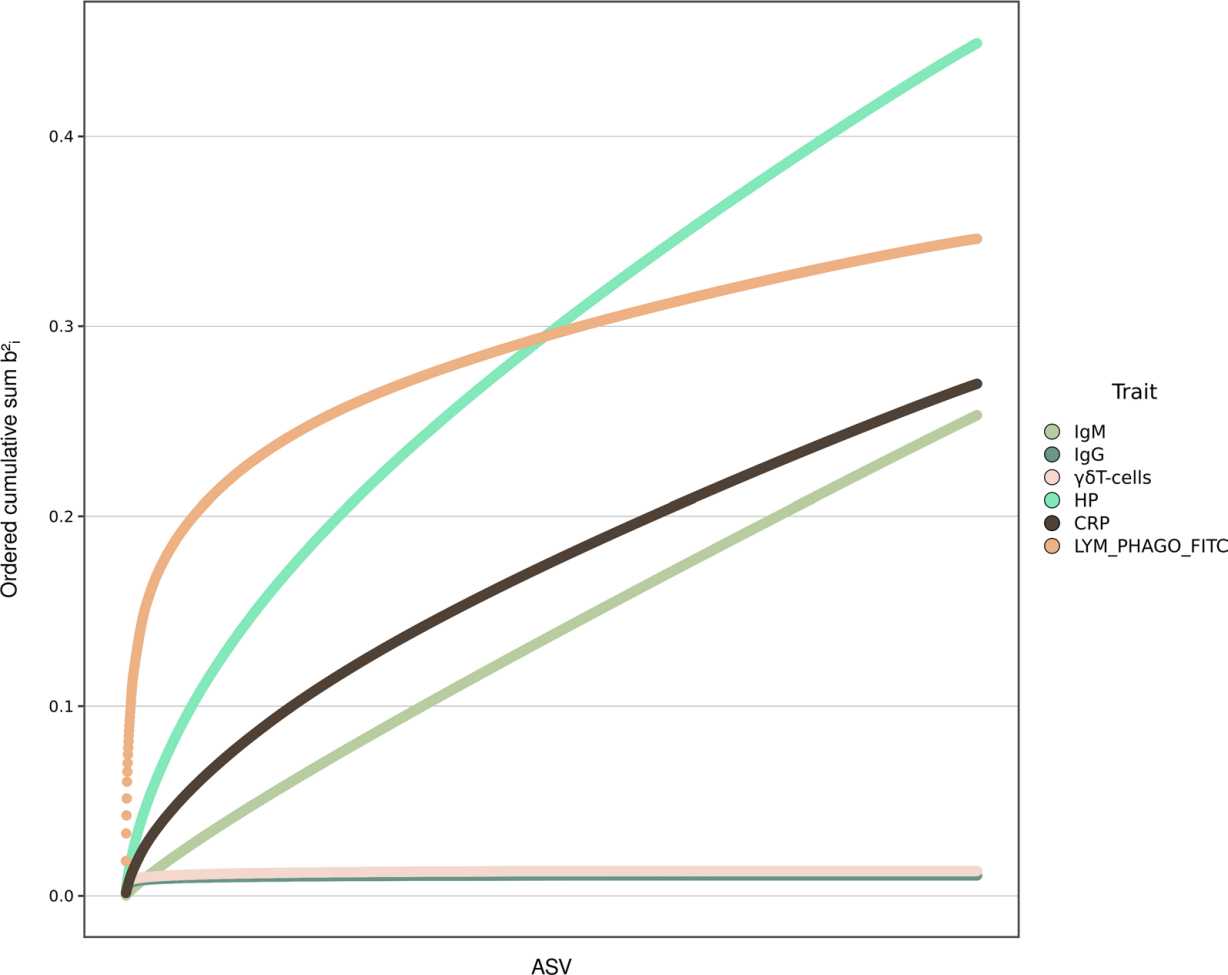
Cumulative sum of the contribution of individual ASV to microbiability for each trait. ASV microbiabilities from the modelling combination that yielded the highest prediction accuracy and that used Bayes C to model microbial abundances

Figure 5 shows the cumulative sum of the contributions of ASV to the phenotypic variance (${\mathrm{b}}_{\mathrm{j}}^{2}$), inferred from a Bayes C analysis. Curves for IgG and percentage of γδ T cells clearly indicated that the contribution of the microbiome was negligible for these traits. As suggested by the straight line, estimates of the effects of ASV on the phenotypes were uniformly distributed for CRP and IgM. For HP, a few ASV had somewhat larger effects, but estimated effects were similar across the other ASV. At the opposite extreme, the cumulative distribution of estimates of effects on LYM_PHAGO_FITC indicated that a few ASV explained a large part of b^2^. For this trait, 19 ASV were responsible for 30% of b^2^ (see Additional file 3: Table S2), i.e., 10% of the total phenotypic variation of this trait.

The effects of the few ASV that were responsible for a large proportion of b^2^ for LYM_PHAGO_FITC can represent direct or indirect genetic effects. Given the special microbial architecture of this trait (Fig. 5), we explored the heritability of the 19 ASV with the largest contribution (see Additional file 3: Table S2). ASV belonging to the *Bacteroidales* order and to the *Ruminococcaceae* family together explained ~ 5% of the phenotypic variance in LYM_PHAGO_FITC; *Treponema* genera and *Lactobacillales* family were jointly responsible for 3% of the phenotypic variation in LYM_PHAGO_FITC. It is relevant to note that some of these ASV exhibited much higher h^2^ estimates than average (see Additional file 2: Fig. S5). Several ASV that were annotated at the genera level had particularly high h^2^ estimates (*Treponema* sp, h^2^ ~ 0.6; *Streptococcus*, h^2^ ~ 0.5, and (see Additional file 3: Table S2).

## Discussion

### Is it worth using holobiont data to improve prediction?

In a previous simulation study [38], we hypothesized that microbiome data might increase predictive ability of complex phenotypes by up to ~ 50% in an optimistic scenario where h^2^ ~ b^2^ ~ 0.25 with stable microbiota composition and large sample size (~ 1k). In general, the literature reports a positive impact of combining genotype and microbiome data for the prediction of phenotype for complex traits, although results vary according to trait and species (Table 1). The results presented here are broadly consistent with these studies.

**Table 1:** Summary of the results in the literature for the prediction of the phenotype for complex traits using genotype and/or microbiome data

Species	Phenotypes	N	Host genotype	Microbiome	Results	Refs.
Cow	Methane emissions; rumen and blood metabolites; milk production efficiency	1016	120k SNPs	512 OTU	B > X	[15]
Cow	Milk fatty acid content	292	39k SNPs	3055 OTU	XB ~ X	[35]
Cow	Milk acetone and β-hydroxybutyric acid	277	436k SNPs	4226 OTU	B > X	[37]
Rabbit	Feed efficiency; growth	425	Pedigree	963 OTU	XB > X	[18]
Japanese quail	Feed intake; P utilization; body weight gain; feed efficiency	758	4k SNPs	1188 OTU	B > X	[22]
Pig	Daily gain; feed conversion; feed intake	207	52k SNPs	2714 OTU	B > X	[21]
Pig	Meat quality	1123	42k SNPs	1755 OTU	XB > X	[34]
Human	Dietary habits; anthropometric and blood measurements	715	545k SNPs	1.36 M genes (metagenomics)	XB > X	[36]

N: Sample size

B > X: performance using microbiome was larger than with genotypes only; XB > X: holobiont model was better than using genotypes only; XB ~ X: both models performed similarly

The advantage of adding microbiota for prediction, on top of genotype data, will depend on its stability and on the ratio of microbiability to heritability. Ramayo-Caldas et al. [9] reported higher estimates of h^2^ than of b^2^ for concentrations of immunoglobulins G and M and the proportion of γδ T cells in pigs of the same age, while estimates of h^2^ and b^2^ were similar for traits related to innate immunity (HP, CRP, and phagocytic capacity of lymphocytes). Here we show that, by default, the safest choice is the use of a holobiont model rather than partial X or B models. We observe that the best holobiont model outperformed, or as least matched, the best partial model, except for LYM_PHAGO_FITC (Fig. 1). In the case of HP concentration, for which the estimate of microbiability was high, the predictive accuracy increased when microbiome information was added (0.29), compared to using only genotype data (0.04). For other traits, such as IgM and CRP, the increase in predictive accuracy for the $\mathrm{XB}$ vs the $\mathrm{X}$ model was ~ 10 and 30%, respectively. Therefore, although microbiota data generally improves prediction and a holobiont model is recommended, gains can be limited compared to the best-case simulated scenario in [38], which was up to 50%

### Impact of the model used for prediction

While using DNA markers for complex trait prediction has a long history and several statistical models have become widely used (e.g., genomic best linear unbiased prediction (GBLUP)), much less is known on optimum methods to leverage microbiome data. A major reason is that distributions of microbiome abundance are highly leptokurtic and remain non-normal even after usual transformations [33]. In addition, stability of microbial composition is controversial, especially for rare microbes [31]. We and others have argued that there is much room for methodological improvement in this area [38, 63].

We observed that ‘sensitivity’ to modelling, i.e., the variability in predictive accuracies across modelling combinations, differed markedly between traits. Interestingly, this sensitivity increased with microbiability. This can be appreciated in Fig. 1 by comparing the dispersion of combined model predictive accuracies for traits related to adaptative immunity (IgM, IgG and γδ T cells) vs. the innate immunity traits (HP, CRP and LYM_PHAGO_ FITC). Then, unsurprisingly, accuracies of models that included abundances were more variable than those that included only genotypes. All this indicates that modelling of microbial abundances deserves special care. This was observed irrespective of whether Bayes C or RKHS was used, which suggests an important role of prior information. Interestingly, for all traits, ensemble method predictions were consistently better, or at worse very similar, than the average prediction obtained for almost all modelling combinations. Therefore, ensemble methods provide a safe and ‘agnostic’, albeit computationally expensive, approach for prediction—and at the cost of hindering biological interpretation [57].

No model was uniformly best across all traits. In our previous simulation study [38], we observed that Bayes C tended to predict phenotype better than RKHS, although the advantage decreased as the number of causative ASV increased. Here, we found that Bayes C was the best model for three traits: IgG, CRP, and LYM_PHAGO_FITC. This suggests that the number of relevant features is smaller for these three traits than for the other traits, for which an infinitesimal model would fit the data better [58].

Microbial abundances do not only have a skewed distribution, they are also correlated between them. This correlation may arise because they are evolutionarily related and can therefore share the same ecological niche [59], or can form ‘micro communities’ for which abundances of several microorganisms follow similar patterns [60]. Thus, we hypothesized that clustering ASV abundances could result in improved predictions, given that the abundance of clusters would have better distributional properties and would average out the noise of the abundance of individual ASV. Clustering did affect predictions but not always positively. We found that clustering improved prediction in cases when the best microbiome modelling option was RKHS, such as for HP and CRP. In contrast, clustering worsened predictions for LYM_PHAGO_FITC, where Bayes C was the best model. Our hypothesis is that clustering can mask strong microbial signals when there are few causative unrelated bacteria by grouping them with irrelevant ASV. Nevertheless, taking each ASV individually seems the safest default choice.

### Dissecting the influence of microbiota on complex traits

In our previous simulation work [38], we showed that distinguishing between direct and indirect genetic effects mediated by the microbiota on a complex phenotype is difficult. Here, estimates of the h^2^ were low for the abundance of most ASV (see Additional file 2: Fig. S5, median = 0.07). This is consistent with previous studies on the genetic control of host genotype over gut microbial composition in pigs [21, 61] and humans [36], and suggests that indirect genetic ASV-mediated effects should be rare or may not have a large impact on complex phenotypes. However, and as previously reported [18, 34–36], there are traits for which putative causative ASV can in turn be highly heritable. For LYM_PHAGO_FITC, the abundance of a few ASV explained a large part of the variability in that trait (Fig. 5), and some of them exhibited high estimates of h^2^ (see Additional file 3: Table S2). In this case, a partial indirect model cannot be ruled out. Bacteria members of the *Spirochaetaceae, Prevotellaceae* and *Streptococcaceae* families were the main candidates for explaining putative indirect effects, given their contribution to the trait variability and their high heritability estimates. Previous studies also reported high estimates of h^2^ for *Prevotellaceae* and *Streptococcaceae* in the swine gut [61], and for *Prevotellaceae* in the human gut [62]. However, this speculation must be considered with care, given the low prevalence of some ASV and the high error level of individual estimates of heritability. Taken together, our results indicate that more sophisticated statistical analyses are needed to get a better understanding of how direct and microbiome-mediate genetic effects can be dissected [11, 63].

## Conclusions

Microbiota information improves the prediction of phenotypes for immunocompetence traits, especially for traits related to innate immunity. As a result, the best default option is a holobiont model, rather than partial models that use only genotype or microbial data. Care in choosing the statistical model is particularly important for traits for which microbiability is high. Somewhat counter-intuitively, clustering microbial taxa does not necessarily help prediction, but if it does, it is for traits for which most microbial ASV have similar, small effects. The best clustering strategy is trait-specific. Confounding of the estimates of heritability and microbiability for a trait may suggest an indirect effect of host genotype through microbial composition. Evidence in favour of indirect genetic effects seems weak though, more sophisticated statistical models than those used here are warranted to settle this issue.

## Supplementary Information

**Additional file 1:** QIIME2 pipeline used to process and combine the two 16S sequence datasets.

**Additional file 2: Fig. S1**. Evolution of heritability estimates along iterations in an holobiont model in CRP to illustrate convergence of the MCMC chain.** Fig. S2**. PCA for the individual batches and the merged dataset of CLR-transformed ASV abundances.** Fig. S3**. Distribution of reads per sample in the merged 16S sequencing data after quality control.** Fig. S4**. Distribution of CLR-transformed ASV abundances, averaged across samples.** Fig. S5**. Distribution of the ASV heritability estimates.

**Additional file 3: Table S1**. Correlation of the Euclidean distances between samples in each pair of datasets.** Table S2**. ASV responsible for 30% of the estimated b2 under the best predictive model for LYM_PHAGO_FITC.

## Data Availability

The raw sequencing data of host genotype and both batches of 16S gut metagenome employed in this article are available in the NCBI’s short read archive (https://www.ncbi.nlm.nih.gov/sra) BioProject PRJNA608629.
