# Supplementary material for: On the holobiont ‘predictome’ of immunocompetence in pigs
Source: Genet Sel Evol. 2023 May 1;55:29. doi: 10.1186/s12711-023-00803-4 (PMC10150480; doi:10.1186/s12711-023-00803-4)
Supplement: Supplementary file 2 — Additional file 2: Fig. S1. Evolution of heritability estimates along iterations in an holobiont model in CRP to illustrate convergence of the MCMC chain. Fig. S2. PCA for the individual batches and the merged dataset of CLR-transformed ASV abundances. Fig. S3. Distribution of reads per sample in the merged 16S sequencing data after quality control. Fig. S4. Distribution of CLR-transformed ASV abundances, averaged across samples. Fig. S5. Distribution of the ASV heritability estimates. [file 12711_2023_803_MOESM2_ESM.docx]

**Supplementary Figures**


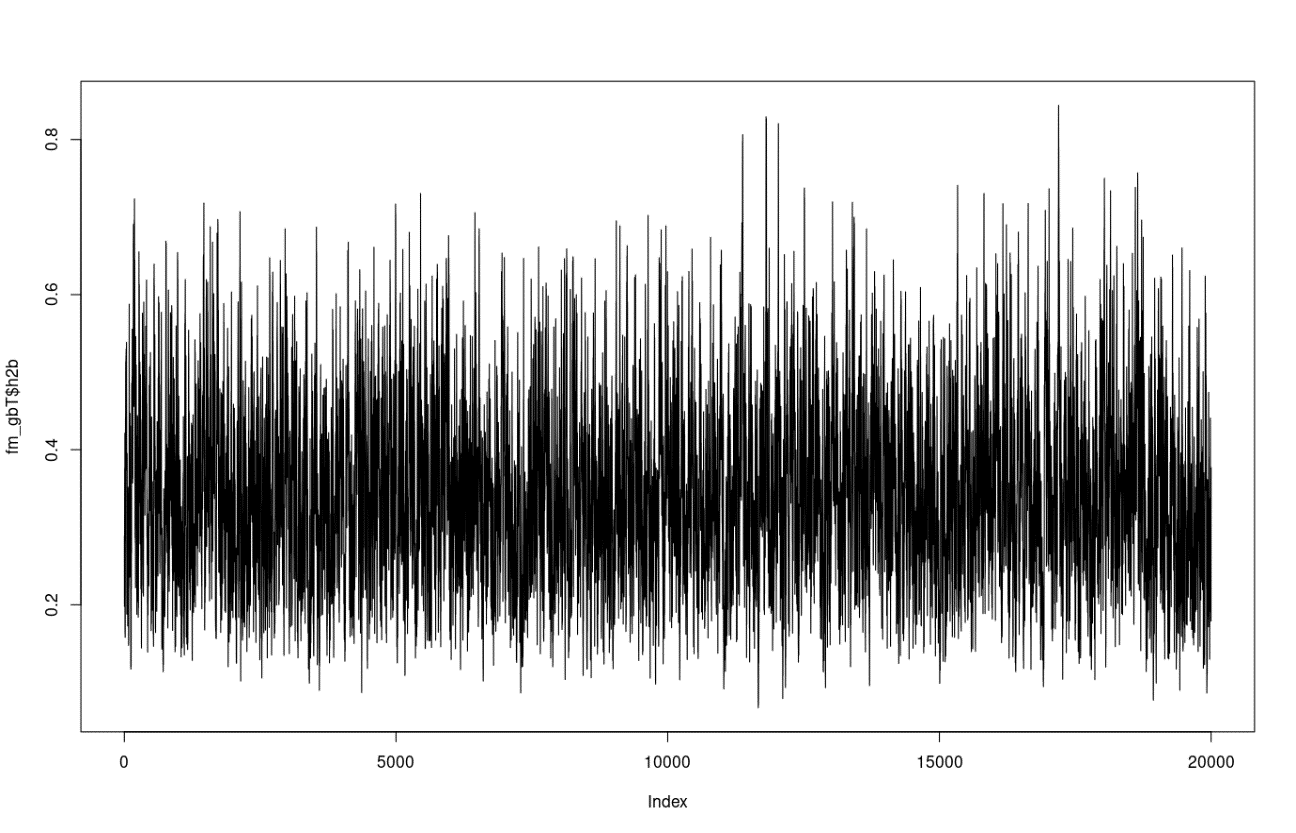


**Supplementary Fig. S1.** Estimated value of LYM_PHAGO_FITC microbiability for each iteration under an holobiont model (genotype + microbiota) solved through RKHS with flat prior.


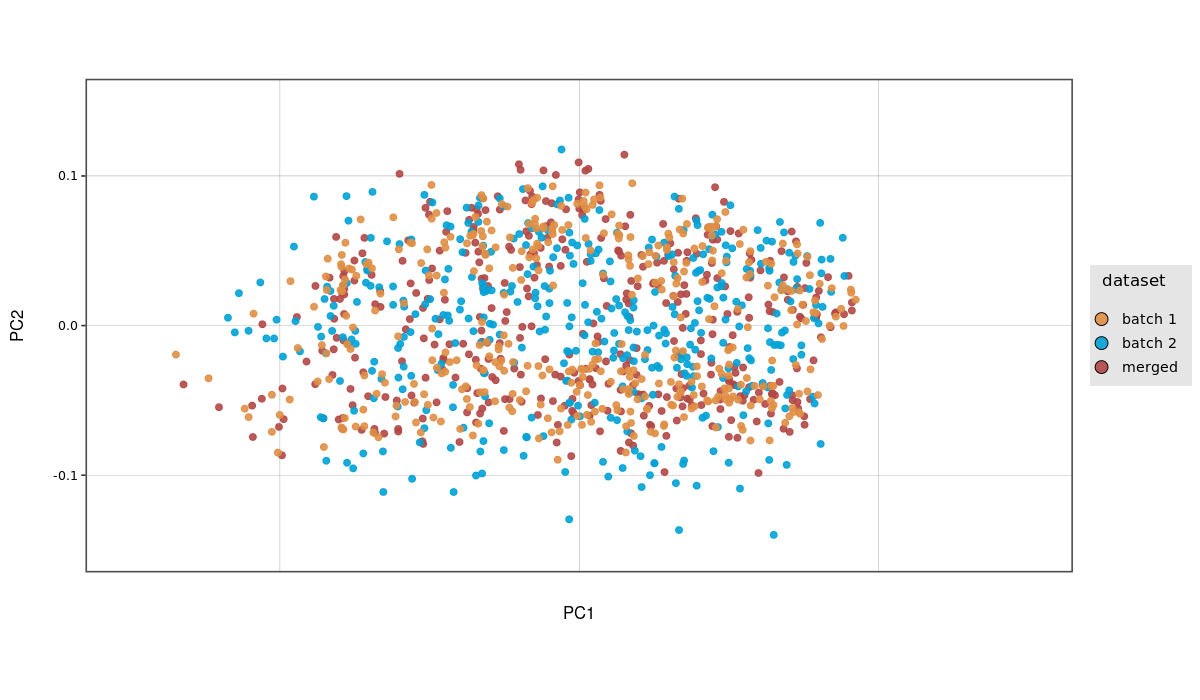


**Supplementary Fig. S2.** PCA for the individual batches and the combined dataset of CLR-transformed ASVs abundances.


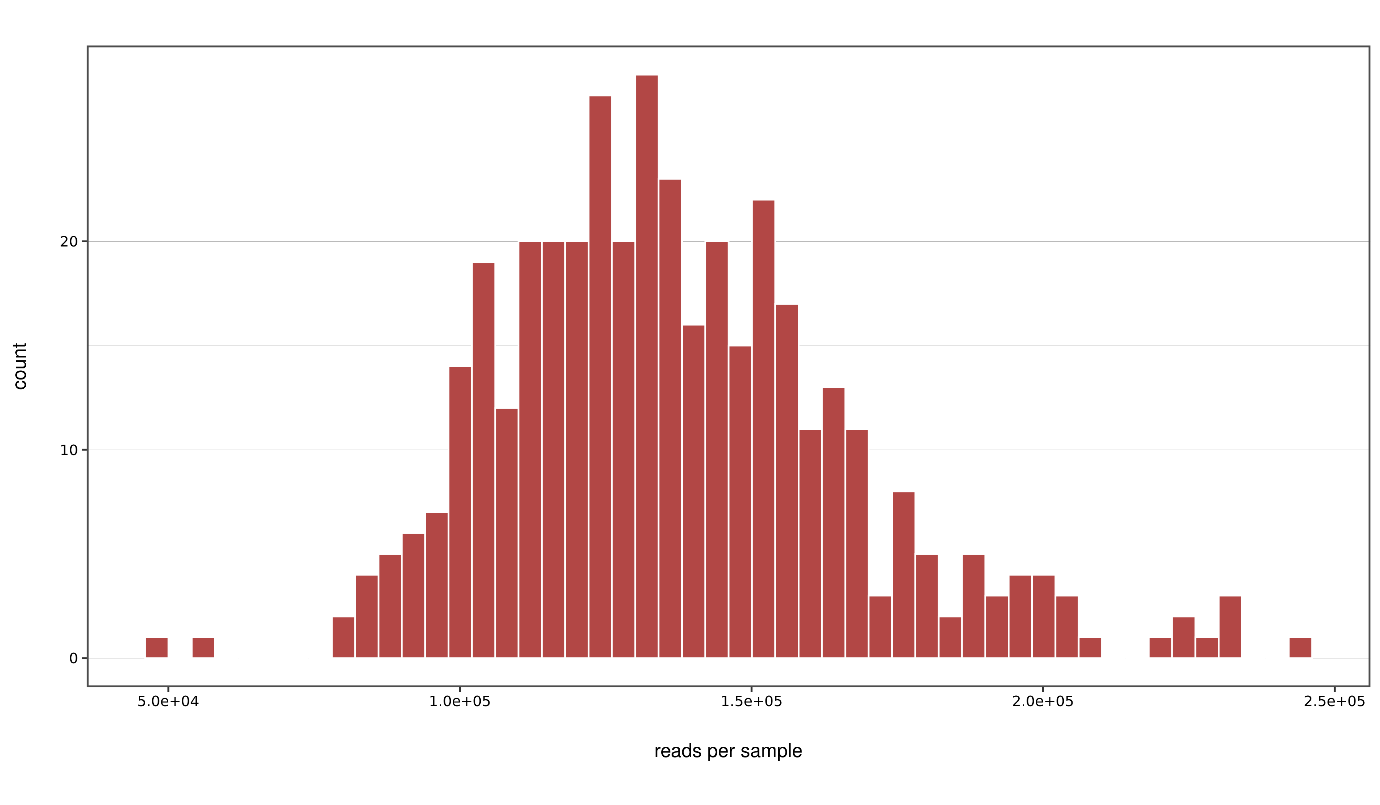


**Supplementary Fig. S3.** Distribution of reads per sample in the combined 16S sequencing data after quality control.


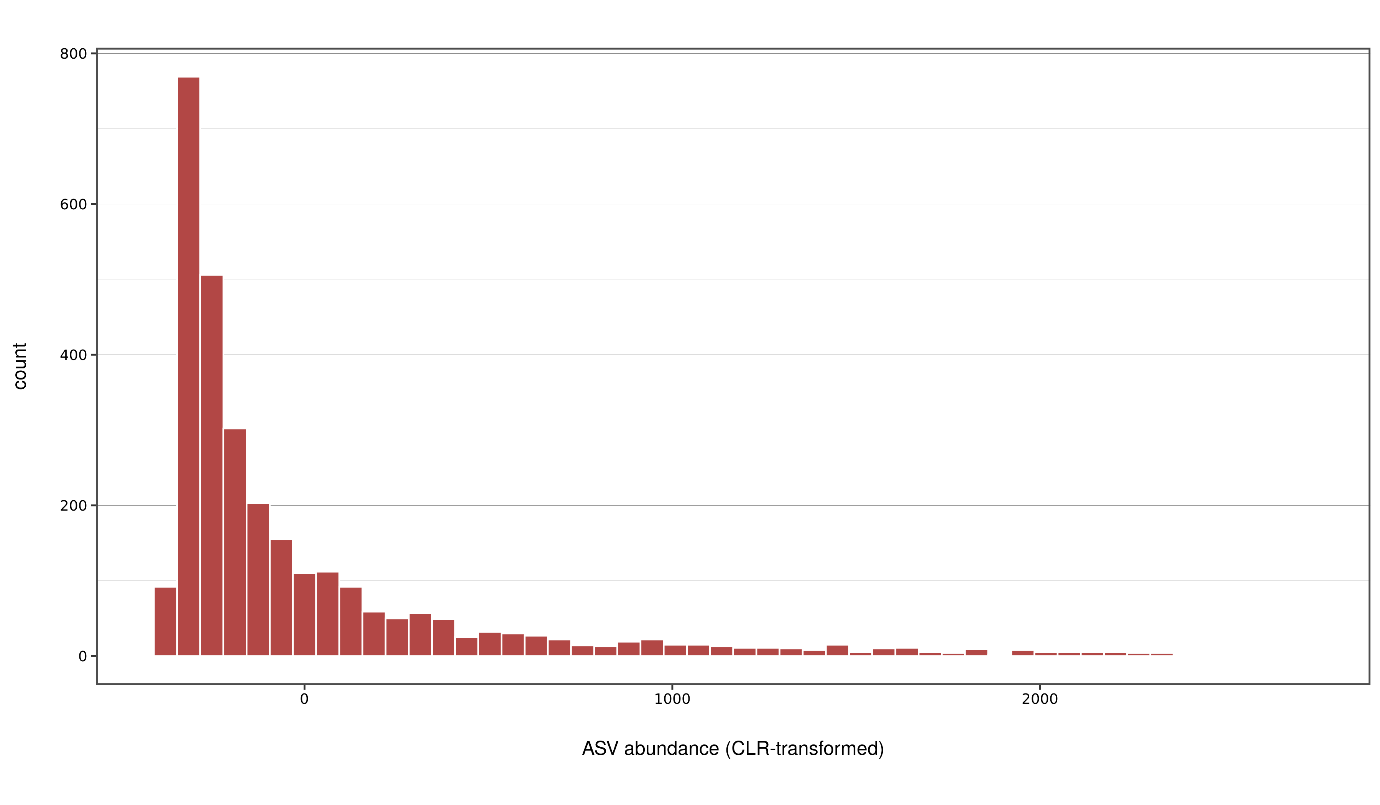


**Supplementary Fig. S4.** Distribution of CLR-transformed ASV abundances averaged across samples.


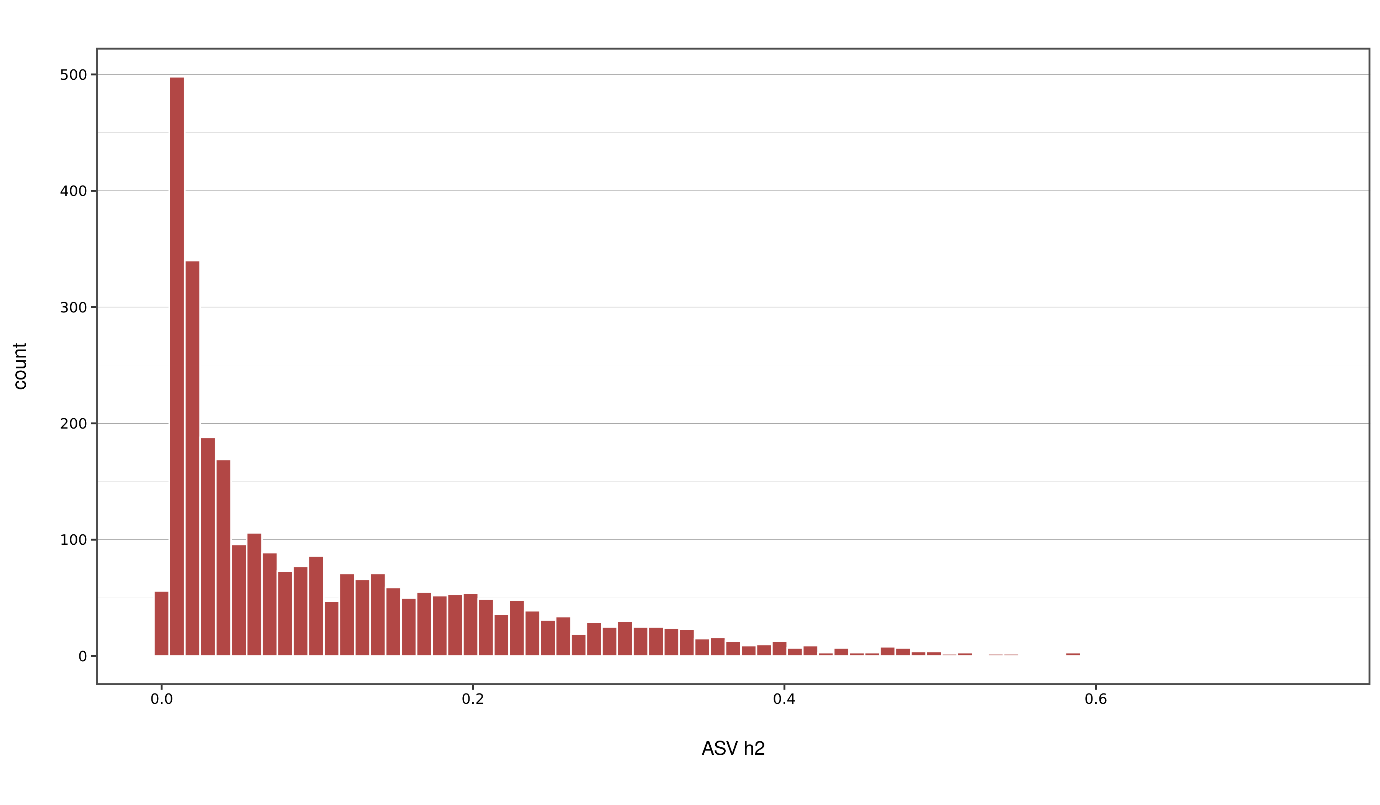


**Supplementary Fig. S5.** Distribution of the ASV heritability estimates.
