# Supplementary material for: On the holobiont ‘predictome’ of immunocompetence in pigs
Source: Genet Sel Evol. 2023 May 1;55:29. doi: 10.1186/s12711-023-00803-4 (PMC10150480; doi:10.1186/s12711-023-00803-4)
Supplement: Supplementary file 3 — Additional file 3: Table S1. Correlation of the Euclidean distances between samples in each pair of datasets. Table S2. ASV responsible for 30% of the estimated b2 under the best predictive model for LYM_PHAGO_FITC. [file 12711_2023_803_MOESM3_ESM.docx]

**Supplementary Tables**

**Supplementary Table S1.** **Correlation of the Euclidean distances between samples in each pair of datasets.**

|  | Batch 1 (NovaSeq) | Batch 2 (MiSeq) |
| --- | --- | --- |
| Batch 2 (MiSeq) | 0.976 | - |
| Merged | 0.999 | 0.982 |

**Supplementary Table S2. ASVs responsible for 30% of the estimated b^2^ under the best predictive model for LYM_PHAGO_FITC.**

| **Order** | **Family** | **Genus** | **Prevalence (%)** | **b_i_^2^­** | **h^2^­** |
| --- | --- | --- | --- | --- | --- |
| *Bacteroidales* | *Prevotellaceae* | *Prevotella* | 2.75 | 0.02±0.01 | 0.32±0.12 |
| *Spirochaetales* | *Spirochaetaceae* | *Treponema* | 10 | 0.01±0.02 | 0.59±0.16 |
| *Clostridiales* | *Ruminococcaceae* | *-* | 4.5 | 0.01±0.01 | 0.04±0.05 |
| *Spirochaetales* | *Spirochaetaceae* | *Treponema* | 11.5 | 0.01±0.01 | 0.61±0.14 |
| *Bacteroidales* | *[Paraprevotellaceae]* | *[Prevotella]* | 88.25 | 0.01±0.01 | 0.08±0.07 |
| *Deferribacterales* | *Deferribacteraceae* | *Mucispirillum* | 51.5 | 0.005±0.01 | 0.06±0.08 |
| *Clostridiales* | *Ruminococcaceae* | *-* | 18.5 | 0.005±0.01 | 0.23±0.11 |
| *Lactobacillales* | *Lactobacillaceae* | *Lactobacillus* | 4 | 0.005±0.01 | 0.01±0.02 |
| *Lactobacillales* | *Streptococcaceae* | *Streptococcus* | 18.5 | 0.004±0.01 | 0.48±0.17 |
| *Bacteroidales* | *[Paraprevotellaceae]* | *[Prevotella]* | 54.25 | 0.003±0.01 | 0.20±0.09 |
| *Clostridiales* | *Clostridiaceae* | *Clostridium* | 3.5 | 0.003±0.01 | 0.003±0.01 |
| *Clostridiales* | *Ruminococcaceae* | *-* | 7.25 | 0.003±0.01 | 0.03±0.05 |
| *Clostridiales* | *Ruminococcaceae* | *-* | 8.25 | 0.003±0.01 | 0.02±0.05 |
| *-* | *-* | *-* | 1.25 | 0.002±0.01 | 0.01±0.02 |
| *Lactobacillales* | *Lactobacillaceae* | *Lactobacillus* | 8 | 0.002±0.01 | 0.12±0.08 |
| *Clostridiales* | *Lachnospiraceae* | *Anaerostipes* | 8.75 | 0.002±0.01 | 0.25±0.13 |
| *Fibrobacterales* | *Fibrobacteraceae* | *Fibrobacter* | 48.5 | 0.002±0.01 | 0.05±0.07 |
| *Bacteroidales* | *[Paraprevotellaceae]* | *[Prevotella]* | 14 | 0.002±0.01 | 0.03±0.04 |
| *Bacteroidales* | *[Paraprevotellaceae]* | *[Prevotella]* | 5 | 0.002±0.01 | 0.29±0.11 |

b_i_², the individual contribution to phenotypic variance; h^2^, the ASV estimated heritability; family and genera assigned by Greengenes 13.8; prevalence, % of samples with presence of each ASV out of 400 total samples.
